# Sources of artifact in measurements of 6mA and 4mC abundance in eukaryotic genomic DNA

**DOI:** 10.1186/s12864-019-5754-6

**Published:** 2019-06-03

**Authors:** Zach K. O’Brown, Konstantinos Boulias, Jie Wang, Simon Yuan Wang, Natasha M. O’Brown, Ziyang Hao, Hiroki Shibuya, Paul-Enguerrand Fady, Yang Shi, Chuan He, Sean G. Megason, Tao Liu, Eric L. Greer

**Affiliations:** 1 Division of Newborn Medicine, Boston Children’s Hospital, 300 Longwood Avenue, Boston, MA 02115 USA; 2 Department of Pediatrics, Harvard Medical School, Boston, MA 02115 USA; 3 Department of Biochemistry and Biostatistics, University at Buffalo Center of Excellence in Bioinformatics & Life Sciences, Buffalo, NY 14203 USA; 4 Department of Neurobiology, Harvard Medical School, Boston, MA 02115 USA; 5 Department of Chemistry and Howard Hughes Medical Institute, The University of Chicago, Chicago, IL 60637 USA; 6 Present address: Department of Chemistry and Molecular Biology, University of Gothenburg, SE-40530 Gothenburg, Sweden; 7 Department of Cell Biology, Harvard Medical School, Boston, MA 02115 USA; 8 Department of Systems Biology, Harvard Medical School, Boston, MA 02115 USA

**Keywords:** DNA epigenome, DNA N6-methyladenosine, 6 mA, DNA N4-methylcytosine, 4mC

## Abstract

**Background:**

Directed DNA methylation on N6-adenine (6mA), N4-cytosine (4mC), and C5-cytosine (5mC) can potentially increase DNA coding capacity and regulate a variety of biological functions. These modifications are relatively abundant in bacteria, occurring in about a percent of all bases of most bacteria. Until recently, 5mC and its oxidized derivatives were thought to be the only directed DNA methylation events in metazoa. New and more sensitive detection techniques (ultra-high performance liquid chromatography coupled with mass spectrometry (UHPLC-ms/ms) and single molecule real-time sequencing (SMRTseq)) have suggested that 6mA and 4mC modifications could be present in a variety of metazoa.

**Results:**

Here, we find that both of these techniques are prone to inaccuracies, which overestimate DNA methylation concentrations in metazoan genomic DNA. Artifacts can arise from methylated bacterial DNA contamination of enzyme preparations used to digest DNA and contaminating bacterial DNA in eukaryotic DNA preparations. Moreover, DNA sonication introduces a novel modified base from 5mC that has a retention time near 4mC that can be confused with 4mC. Our analyses also suggest that SMRTseq systematically overestimates 4mC in prokaryotic and eukaryotic DNA and 6mA in DNA samples in which it is rare. Using UHPLC-ms/ms designed to minimize and subtract artifacts, we find low to undetectable levels of 4mC and 6mA in genomes of representative worms, insects, amphibians, birds, rodents and primates under normal growth conditions. We also find that mammalian cells incorporate exogenous methylated nucleosides into their genome, suggesting that a portion of 6mA modifications could derive from incorporation of nucleosides from bacteria in food or microbiota. However, gDNA samples from gnotobiotic mouse tissues found rare (0.9–3.7 ppm) 6mA modifications above background.

**Conclusions:**

Altogether these data demonstrate that 6mA and 4mC are rarer in metazoa than previously reported, and highlight the importance of careful sample preparation and measurement, and need for more accurate sequencing techniques.

**Electronic supplementary material:**

The online version of this article (10.1186/s12864-019-5754-6) contains supplementary material, which is available to authorized users.

## Background

Directed DNA methylation by specific methyltransferase enzymes occurs in both prokaryotes and eukaryotes. These modifications can mark regions of the genome for control of a variety of processes, including base pairing, duplex stability, replication, repair, transcription, nucleosome positioning, X-chromosome inactivation, imprinting, and epigenetic memory [1–3]. The most well studied and abundant DNA methylation in eukaryotes is 5mC, a mark typically associated with repressed chromatin that occurs on ~ 3–8% of cytosines in mammals [4]. In bacteria, directed DNA methylation on N6 of adenine (6mA) regulates mismatch repair, DNA replication and transcription. 4mC has been identified in thermophilic bacteria and archaea, and to a lesser extent in mesophilic bacteria [5–9]. Relatively little is known about the function of 4mC beyond its role in the restriction-modification system, which 6mA also regulates. 6mA, which was previously thought to be restricted to unicellular organisms, was recently reported to occur in multicellular organisms including fungi, Arabidopsis, worms, flies, frogs, zebrafish, and mammals, but its function remains unclear [10–22]. Some reports have suggested that 6mA is regulated in metazoa in response to stress [18, 19, 23], development [11, 14, 15], or cancer [24], findings which suggest a functional role. However, other groups have been unable to detect 6mA in metazoa [25–27], raising the question of whether detection techniques are reliable.

The application of highly sensitive detection and quantification methods, including single molecule real time sequencing (SMRTseq) [28] and ultra-high performance liquid chromatography coupled with mass spectrometry (UHPLC-ms/ms), has led to widely varying estimates of 6mA abundance that range from 1 to 8000 ppm (ppm)(0.0001–0.8%) in various eukaryotic genomes [10–22, 29]. SMRTseq measures the kinetic rate at which each new base is incorporated, which is altered by DNA modifications [28]. SMRTseq analyses has been used by one group to map methylation sites genome-wide in seven eukaryotic genomes, including mammals, and identify putative 4mC and 6mA sites [30]. However, UHPLC-ms/ms performed by another group did not detect 4mC or 6mA in human cells, mouse cells or tissues [25]. Here, we compare SMRTseq quantification to UHPLC-ms/ms measurements and find that while SMRTseq is relatively accurate in organisms with abundant 6mA, the accuracy of SMRTseq declines as 6mA becomes rarer. We additionally find that detection of 4mC by SMRTseq is unreliable in the organisms we examined. Although UHPLC-ms/ms is more accurate and sensitive for quantifying DNA methylation if samples are prepared without contamination, machine to machine variations in detection limits could complicate comparisons. In addition, we have found that the commercial enzymes used for digesting DNA to nucleosides before UHPLC-ms/ms analysis can be contaminated with methylated DNA, which can be misinterpreted as evidence of methylation, highlighting the need for mock digested experiments as controls. Here, we performed an analysis of 4mC and 6mA in 2 prokaryotic and 16 eukaryotic genomes using a UHPLC-ms/ms method designed to take into account sources of contamination. We identified 6mA at concentrations previously reported in unicellular organisms [12, 31–33], but found that in general 6mA occurs rarely in the genomes of all metazoans tested under normal growth conditions and that 4mC generally falls below our limit of detection. However, 6mA concentrations of the exact same DNA samples, calculated around the limit of detection, were variable from machine to machine. Additionally, we have found that sonication of DNA can lead to artifactual chromatogram peaks, which masquerade as 4mC. We also confirmed previous reports [25, 34] that mammalian cells can incorporate exogenous methylated DNA bases into their genomes, raising the possibility that some detected 6mA and 4mC might be due to random incorporation of bacterial methylated nucleosides rather than directed DNA methylation. Together our results suggest that 4mC and 6mA are present at fold lower levels, in metazoan gDNA than we and others previously reported; in some species its presence in genomic DNA under normal growth conditions is at or below our detection limits. Improved sample preparation and detection technologies along with biological situations where DNA methylation becomes elevated are needed to evaluate the extent and biological role of these rare DNA methylation events.

## Results

### Enzymes used to digest DNA for quantification contain unmethylated and methylated DNA

**Fig. 1:**
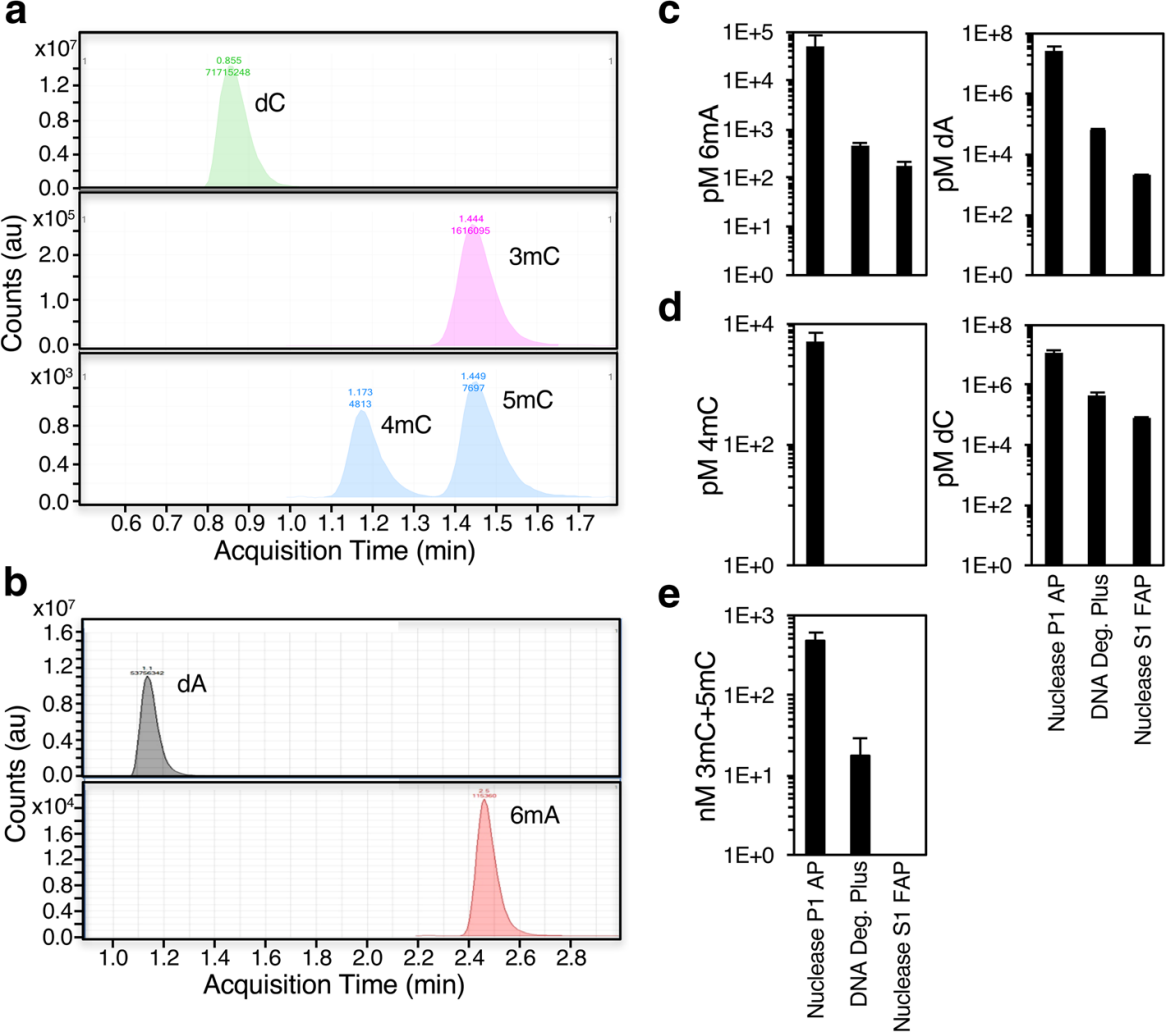
Purified nucleases and phosphatases contain methylated DNA. **a** UHPLC-ms/ms chromatography peaks of nucleoside standards corresponding to unmodified deoxycytidine (dC) and methylated deoxycytidines (3mC, 4mC or 5mC). **b** UHPLC-ms/ms chromatography peaks of nucleoside standards corresponding to unmodified deoxadenosine (dA) and N6-methylated deoxyadenosine (6mA). **c** Calculated molarity of 6mA (left panel) and adenine (right panel) in the three enzyme mixes: 1) Nuclease P1 mix (Nuclease P1, Phosphodiesterase 1, and alkaline phosphatase), 2) DNA degradase Plus, and 3) Nuclease S1 mix (Nuclease S1 and Fast alkaline phosphatase) reveals that the Nuclease P1 mix is more heavily contaminated than DNA degradase or Nuclease S1 mix. **d** Molarity of 4mC (left panel) and cytosine (right panel) in the three enzyme mixes shows that Nuclease P1 mix is more heavily contaminated than other digestion mixes. **e** Molarity of 3mC and 5mC in the three enzyme mixes shows that Nuclease P1 mix is more heavily contaminated than other digestion mixes. Each bar represents the mean +/− standard error of the mean for 3–10 independent mock reactions

Because 6mA has been reported to occur in a variety of metazoan genomes by some groups [10–22], but not by others [25–27], we developed a UHPLC-ms/ms method that can distinguish 5mC or 3mC, 4mC, and 6mA. The UHPLC-ms/ms buffers and flow rates were optimized to separate methylated nucleosides using pre-methylated standards (Fig. 1a and b). Before UHPLC-ms/ms analysis, gDNA must first be digested to individual nucleosides. This is accomplished by treatment with a nuclease to cleave the chains of nucleotides to individual bases and an alkaline phosphatase to reduce the bases to nucleosides. Because bacteria have relatively high levels of DNA methylation, and the enzymes used to digest gDNA are purified from bacteria, we were concerned about possible DNA contamination of the digestion enzymes. We carefully tested three commercially available nucleases and phosphatases: 1) Nuclease P1 purified from the fungus *Penicillium citrinum* and Phosphodiesterase I from *Crotalus adamanteus* venom with *E. coli* alkaline phosphatase; 2) Nuclease S1 purified from the fungus *Aspergillus oryzae* with *E. coli* purified fast alkaline phosphatase; and 3) DNA degradase plus, which contains an unidentified nuclease and an unidentified alkaline phosphatase. The enzyme mixes contained a wide range of contaminating DNA - 2 nanomolar to 25 micromolar adenine and 78 nanomolar to 12 micromolar cytosine (Fig. 1c and d, right panels). Methylated bases were also detected in all the enzyme preparations to varying degrees. Nuclease P1 with Phosphodiesterase I and alkaline phosphatase contained the highest amounts of both methylated and unmethylated nucleosides. It was contaminated with micromolar concentrations of adenine and cytosine and 50 nanomolar 6mA, 495 nanomolar 5mC or 3mC, and 5 nanomolar 4mC, while DNA degradase plus or Nuclease S1 with fast alkaline phosphatase contained two logs less 6mA and 5mC and no detectable 4mC (Fig. 1c-e; DNA degradase plus: 0.5 nanomolar 6mA, 18 nanomolar 5mC or 3mC, Nuclease S1 mix: 0.2 nanomolar 6mA, no detectable 5mC or 3mC). Together these results suggest that the enzymes used to digest DNA are contaminated with unmethylated and methylated DNA nucleosides. In all subsequent experiments, we used DNA degradase plus, and analyzed a mock digestion water sample in parallel with gDNA samples to set a background level of DNA methylation, which was subtracted from each sample.

### Quantification of 6mA and 5mC in DNA from 16 eukaryotic species

**Fig. 2:**
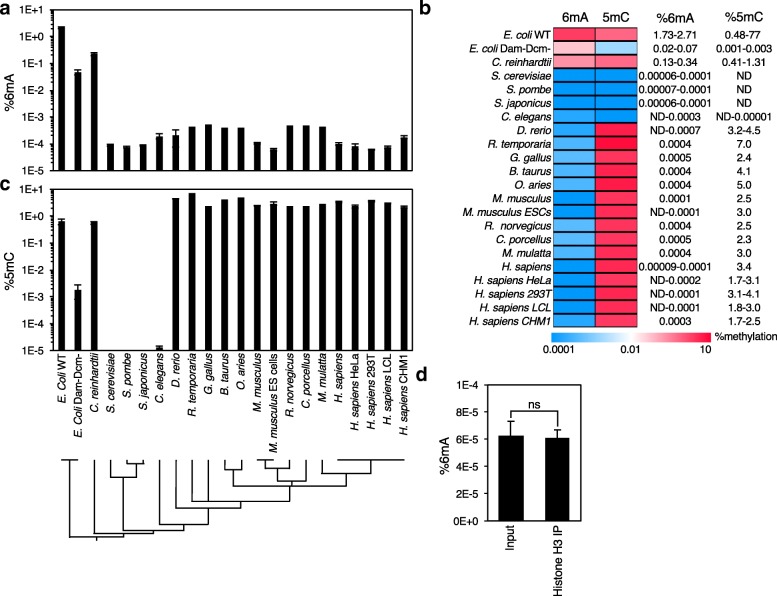
UHPLC-ms/ms quantification of 6mA and 5mC DNA methylation in eukaryotes. UHPLC-ms/ms quantification of **a** 6mA and **c** 5mC in 16 eukaryotic species and 2 bacterial strains. Phylogram displayed below represents the evolutionary distance between species. Non-gnotobiotic mammalian and *G. gallus* samples were extracted from brains, *R. temporaria* samples were extracted from liver, *D. rerio* samples were extracted from the posterior end of adults, *C. elegans* were extracted from bleached embryos or young adults, the *E. coli* represent two different K12 strains; wild-type OP501 and *dam*^−^*dcm*^−^ (NEB C2925). Each bar represents the mean +/− standard error of the mean for 2–20 independent samples except for single samples for *R. temporaria, G. gallus, O. aries, R. norvegicus, C. porcellus, B. Taurus, and M. mulatta*. We note that in several UHPLC-ms/ms experiments 6mA was below our limit of detection (< 0.00005%) in metazoan DNAs (data not shown). **b** A heat map of the 6mA and 5mC quantifications and calculated values demonstrates 6mA is rare, if present at all, relative to 5mC in metazoan. **d** Chromatin immunoprecipitation of 293 T gDNA with a histone H3 antibody shows no significant depletion of 6mA levels as assessed by UHPLC-ms/ms. Each bar represents the mean +/− SEM of 3 independent experiments of 1–3 replicates. ns: not significant as assessed by Welch’s t test

UHPLC-ms/ms with background corrections was used to examine 4mC, 5mC, and 6mA concentrations in gDNA samples from eukaryotic gDNA samples from 16 diverse eukaryotic species, including representative protists, worms, insects, amphibians, birds, rodents and primates (Fig. 2). The frequency of methylated nucleosides in eukaryotic gDNA was compared to the frequency in *E. coli* and in *dam*^*−*^*dcm*^*−*^ *E. coli* deficient in the DNA adenine methyltransferase (Dam) and DNA cytosine methyltransferase (Dcm) enzymes [35], which are responsible for most 6mA and 5mC modifications in bacteria, respectively. As expected, in WT *E. coli* we detected relatively high levels of 6mA (1.73–2.71%) and somewhat less 5mC (0.48–0.77%), as has been previously reported [36]. Similarly, deletion of the predominant 6mA and 5mC methyltransferases caused a several log fold reduction in detected methylated bases (0.02–0.07 6mA and 0.001–0.003% 5mC), suggesting that our UHPLC-ms/ms measurements are accurate. The protist *Chlamydomonas reinhardii* had relatively high levels of 6 mA (0.13–0.34%), as has been previously reported [32, 33], but almost all metazoan gDNAs that we examined had very low levels of 6mA (0.3 to 4 ppm, 0.00003–0.0004%), or were below our limit of detection (Fig. 2a-c), consistent with a recently published report [25]. Detection of 6mA or its absence varied from experiment to experiment (Additional file 1 a and b), even on the same samples measured at independent times on the same or on different UHPLC-ms/ms machines. Although we made every effort to purify eukaryotic samples without prokaryotic DNA contamination (Additional file 2 a), it was virtually impossible to achieve complete purity. Most metazoan DNA samples contained 0.1–2% prokaryotic DNA as assessed by quantitative RT-PCR using 16S rRNA primers (Additional file 2 a). Of eukaryotic samples that contained less than 2% prokaryotic DNA contamination, there was no correlation between prokaryotic DNA contamination and apparent DNA methylation levels (R^2^ = 0.08859, *p* = 0.32). Because 16S copy number varies between prokaryotes [37] and different prokaryotes have different levels of DNA methylation [6], it is difficult to assess how much prokaryotic DNA methylation contamination contributes to the values obtained for eukaryotic DNA.

To eliminate as much as possible sources of bacterial contaminating DNA, we analyzed gDNA from brains of germ-free gnotobiotic mice. When detected, 6mA levels in the gnotobiotic brain DNA, although low, were comparable to levels in other gnotobiotic tissues, non-gnotobiotic mouse brains, mouse ES cells and other metazoan samples (Fig. 2a, b, and Additional file 3), suggesting that the 6mA DNA methylation detected after subtracting the background is in fact of eukaryotic origin. To examine further whether the detected signal was derived from eukaryotic DNA, we measured 6mA only on chromatinized eukaryotic DNA from human kidney 293 T cells isolated by chromatin immunoprecipitation (ChIP) using anti-histone H3 by UHPLC-ms/ms. The proportion of 6mA in isolated chromatin DNA was not statistically different from input DNA (Fig. 2d and Additional file 1 d). These results, taken together, suggest that most metazoan species have rare 6mA modifications to gDNA in the ppm range under basal conditions. However, because the values we obtained are either not significantly above background or just above background, we cannot completely rule out the possibility that 6mA measurements in all the eukaryotic species we examined under basal conditions, with the exception of *C. reinhardtii*, are an artifact. More sensitive detection techniques or elimination of bacterial DNA contamination from enzyme preparations and eukaryotic DNA samples will be required to settle this question with confidence. We believe proper sample preparation and measurement, including measuring mycoplasma level or bacterial contamination in every biological sample, are required in future reports of 6mA in metazoan. With these precautions taken, identification of specific cell populations or biological conditions where 6mA is significantly elevated, under these stringent analysis parameters, will lend additional credence to the existence and significance of 6mA in metazoan genomic DNA.

The UHPLC-ms/ms method readily detected the more abundant methylated base 5mC in genomic DNA samples, although it couldn’t distinguish 5mC from 3mC since both elute at the same retention time (Fig. 1a). 5mC (+ 3mC) levels ranged from between 1.7–7% in all of the more recently evolved eukaryotes examined, but was not detected in three yeast species, *S. pombe, S. cerevisiae, and S. japonicus,* as previously reported [3] (Fig. 2b). We detected very low levels of 5mC and/or 3mC in *C. elegans* (0.000014%). These low levels of DNA methylation in *C. elegans* could reflect the DNA damage mark 3mC, rather than 5mC. These results confirm that 5mC is the predominant DNA methylation mark in more recently evolved eukaryotes.

### Detection of a sonication-induced and 5mC-dependent methylated cytosine variant in metazoan DNAs

**Fig. 3:**
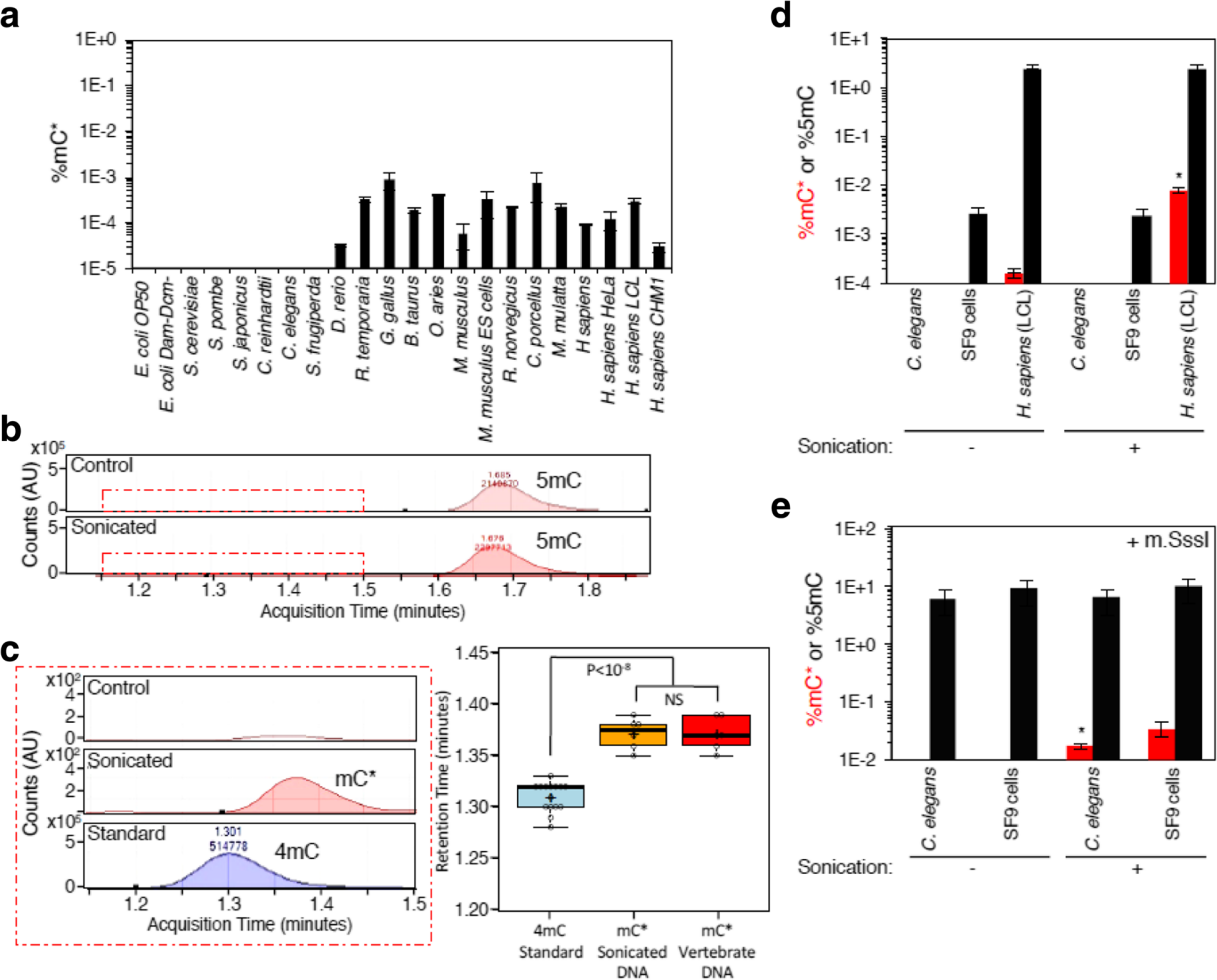
Sonication of DNA generates a 5mC-dependent methylcytosine peak. **a** A methylcytosine peak, denoted mC*, was detected in the DNAs of eukaryotes which have high levels of 5mC (vertebrates and mammals) but undetectable in other organisms. Each bar represents the mean +/− standard error of the mean for 2–13 independent samples. **b** Representative UHPLC-ms/ms chromatograms displays the acquisition time of 5mC before and after sonication of human lymphoblastoid cell line (hLCL) genomic DNA. **c** mC* is detected at a later acquisition time than 4mC. Left panel depicts a zoomed in examination of hLCL genomic DNA with or without sonication from b) demonstrates a peak that appears at a later acquisition time than 4mC standard (lower panel). The inset displays boxplots of the distribution of retention times for 4mC standards (*n* = 15), mC* from DNA sonication of gDNA from several independent eukaryotic species (*n* = 6) and mC* detected in un-sonicated 5mC-containing DNAs from the same samples (n = 6). **d** Sonication of human DNA from a lymphoblastoid cell line (LCL), but not DNA from SF9 insect cells or *C. elegans* results in the generation of mC*. %mC* is shown in red and %5mC is shown in black. This graph represents the mean +/− standard error of the mean for 2 independent experiments. **e** Methylation of *C. elegans* or SF9 cell genomic DNA with the CpG C5-methyltransferase m.SssI followed by sonication is sufficient to generate mC*. %mC* is shown in red and %5mC is shown in black. This graph represents the mean +/− standard error of the mean for 2 independent experiments. *: *p* = 0.0136 by unpaired t test

We next quantified 4mC in WT and *dam*^*−*^*dcm*^*−*^ *E. coli* and the same 16 diverse eukaryote species using our UHPLC-ms/ms method. The enzyme preparations had no 4mC contamination (Fig. 1) and as expected [6] the bacterial DNA samples also had no detectable 4mC (Fig. 3a). A small, but well-defined peak with a chromatographic mobility close to where 4mC standards ran was detected in more recently evolved eukaryotic species such as *D. rerio, R. temporaria, G. gallus, B. Taurus, O.aries, M.musculus, R. novegicus, C. porcellus, M.mulata,* and *H. sapiens*, but not in *E. coli* or any of the more ancient eukaryotes, including the three yeast species, *C. reinhardii, C. elegans,* or *S. frugiperda*. This peak consistently eluted 0.04–0.05 min after the 4mC standards (12 trials; *p* < 0.00001 Mann-Whitney U test, Fig. 3b and c). Because we were uncertain about the origin of this peak, we designated it mC*. mC* was only detected in organisms that have elevated levels of 5mC, and its abundance increased after sonication (Fig. 3d). Because mC* was present in organisms which contained 5mC and was enriched upon sonication we hypothesized that mC* was a sonication byproduct of 5mC-containing DNA. To test this hypothesis, we purified gDNA from *C. elegans* and SF9 insect cells, both of which had very low to undetectable concentrations of 5mC, and treated them with the C5-cytosine methyltransferase m.SssI, which C5-methylates all CpG dinucleotides [38]. Sonicated m.SssI-treated *C. elegans* or SF9 genomic DNA, but not untreated or unsonicated DNA, contained detectable mC* (Fig. 3e). This sonication-induced peak required the presence of polynucleotides, as sonication of a mixture of free 5mC nucleotides and dNTPs did not generate detectable mC* (data not shown). Together, these results suggest that sonication of DNA samples that contain 5mC generates a methylcytosine variant distinct from 4mC and that 4mC was not detectable in the eukaryotic species we examined. The molecular identity of this mC* remains to be determined; whether it exists in vivo or is just a sonication artifact is unclear.

### Bacteria adhering to zebrafish chorion can appear as a developmentally regulated 6mA and 4mC artifact

**Fig. 4:**
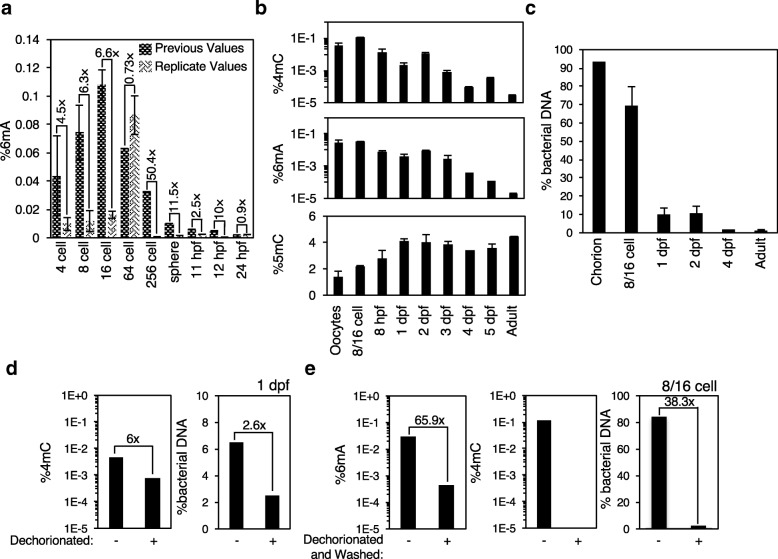
Bacteria adhering to zebrafish chorion presents as a developmental change in 6mA and 4mC concentrations. **a** Replicate UHPLC-ms/ms quantification of zebrafish displays some change in 6mA quantification relative to previously reported [14] values but the developmental decrease was reproduced. Previous values are displayed in trellis bars and new values are displayed in weave bars. Each bar represents the mean +/− standard error of independent samples. **b** Initial UHPLC-ms/ms quantification of zebrafish displays a developmentally correlated increase in 5mC and decrease in 4mC and 6mA. hpf = hours post-fertilizatoin, dpf = days post-fertilization. Each bar represents the mean +/− standard error of the mean of 2–4 independent samples. **c** % bacterial DNA decreases across development as assessed by real-time RT PCR using prokaryote-specific 16S rRNA and zebrafish specific *frrs1b* primers. There is a significant decline in bacterial content with time as assessed by one-way ANOVA (*p* < 0.0001). **d** Dechorionation of 1 dpf zebrafish embryos causes a 6-fold decrease in 4mC (left panel) and 2.6-fold decrease in bacterial contamination (right panel) as assessed by UHPLC-ms/ms and real-time RT PCR respectively. **e** Dechorionation followed by 70% ethanol washing causes a 65.9-fold decrease in 6mA (left panel), elimination of detectable 4mC signal (middle panel), and a 38.3-fold decrease in bacterial contamination (right panel) as assessed by UHPLC-ms/ms and real-time RT PCR

6mA has been reported as a developmentally regulated DNA modification in *Drosophila,* zebrafish, pigs, and Arabidopsis [11, 14, 15]. Because of the low to undetectable levels of 4mC and 6mA we found in eukaryotes, including gDNA from adult *D. rerio*, under basal conditions using UHPLC-ms/ms (Figs. 2 and 3), we next examined 4mC, 5mC, and 6mA abundance during early zebrafish development using the same method. Zebrafish have been reported to display increasing levels of 5mC and decreasing levels of 6mA during development [14, 39]. We first remeasured 6mA values across a developmental gradient and found 6mA levels were slightly lower than we had previously reported, but that the developmental decrease in 6mA replicated (Fig. 4a). To examine this more thoroughly, we measured DNA methylation levels in a separate lab on independently housed zebrafish. We found that 5mC levels increased (*p* = 0.001 by one-way ANOVA), and that 4mC and 6mA were relatively abundant in early embryos and declined as development proceeded (Fig. 4b, 4mC: *p* < 0.0001, 6mA: *p* = 0.1474 by one-way ANOVA). However, the relative abundance of prokaryotic DNA content, assessed by 16S rRNA and *frrs1b* (a zebrafish gene) specific primers, also declined as development proceeded (Fig. 4c, p < 0.0001 by one-way ANOVA). We had previously performed 6mA immunoprecipitation followed by sequencing to examine how 6mA localization changed throughout zebrafish development [14]. This sequencing data revealed low levels of mapped bacterial DNA sequence in addition to unmapped reads which did not display a developmental decline (Additional file 2 b). The bacterial DNA content did increase in these sequencing samples after IP (Additional file 2 b), however, these reads were excluded from the sequencing analysis. The chorion that encases developing zebrafish from fertilization until 3 days post-fertilization (dpf) is exposed to diverse microbial species in food and zebrafish feces [40], and therefore we hypothesized that the chorion could be a source of sample contamination in a developmental timing specific manner. Indeed, dechorionation alone, or in combination with 70% ethanol washing, eliminated the majority of bacterial DNA contamination and with it, the majority of 4mC and 6mA signals (Fig. 4d and e). Together these data suggest that 5mC increases during zebrafish development, consistent with previous work [39], but most of the observed changes in 4mC and a good portion of 6mA during development could be due to bacterial contamination of embryo samples. Our previous work had detected a developmental decrease in 6mA concentrations by immunofluorescence (IF) and by 6mA-IP sequencing [14]. Therefore, if IF and 6mA-IP sequencing are accurate, 6mA could still decrease across early development. Due to the contamination of cultured zebrafish, we cannot confidently and precisely quantify DNA methylation levels at specific stages. The development of more quantitative sequencing methods, equivalent to bisulfite sequencing for detection of 5mC, are required to thoroughly analyze the dynamics of 6mA during early development. We believe that more mechanistic studies, including the manipulation of potential 6mA methyltransferases and demethylases, are required to thoroughly dissect the potential roles of 6mA at early development and to support the IF and IP results which can be prone to false positive signals [41, 42].

### SMRTseq overestimates 4mC in bacteria

An accurate sequencing method that distinguishes methylated from unmethylated residues should distinguish bona fide methylation in animal genomes from spurious detection due to bacterial contamination. SMRTseq takes advantage of the effects of base modifications in the DNA template on the kinetics of incorporation of complementary nucleotides, to infer DNA methylation events [28]. This technique has been validated in a number of unicellular species [30, 31, 43–46] and we and others, used SMRTseq to map 6mA events in metazoa [10, 13, 15, 20, 30, 47]. SMRTseq is appealing because it provides an independent method for validating the presence of 6mA and can identify modified bases in their sequence context at nucleotide resolution. However, SMRTseq also has a relatively low signal-to-noise ratio and a relatively high false positive rate. In prokaryotes and protists methylation occurs consistently at the same sequence sites. However, if methylation events are less consistent or if the frequency of a base modification is close to the detection level, the error rate can be high. To test whether SMRTseq is sensitive enough to detect rare methylation events, we first analyzed SMRTseq datasets we generated for WT and *dam*^−^*dcm*^−^ *E. coli,* which have lower levels of 5mC and 6mA (Fig. 2)*.* SMRTseq analysis identified 1.3% of the adenines as 6mA and 6.0% of the cytosines as 4mC in WT *E. coli*. Paired UHPLC-ms/ms quantification of WT *E. coli* detected 2.4% 6mA, in the same range as SMRTseq, but 4mC was below the limit of detection (< 0.00005%). This large discrepancy between 4mC values suggests that SMRTseq is incorrectly calling 4mC peaks. GATC is the motif recognized by the Dam methylase [48], and 92% of the 6mA sites identified in WT *E. coli* were present in GATC motifs. In *dam*^−^*dcm*^−^ *E. coli*, SMRTseq identified 0.14% of the adenines as 6mA, a 27-fold reduction compared to WT. In *dam*^−^*dcm*^−^ *E. coli*, there was no reduction in 4mC content - 7.1% of the cytosines were 4mC, which was similar to the WT value of 6.0%. These data suggest that SMRTseq has a high false positive rate for detecting 4mC. By contrast, and as expected, UHPLC-ms/ms quantification of 6mA in *dam*^−^*dcm*^−^ *E. coli* DNA was significantly reduced in the mutant compared to WT bacteria to 0.04% 6mA (60-fold reduction) and 4mC remained undetectable (Table 1). Together these data suggest that 6mA identification by SMRTseq in prokaryotes appears to be reliable, while 4mC identification by SMRTseq, using standard algorithms, exceeds levels measured using UHPLC-ms/ms by many orders of magnitude and may be unreliable. Several cytosine modifications alter the polymerase kinetics in SMRTseq [49], making it difficult for this technology to correctly assign N4-cytosine methylation. For appropriate identification of 4mC it will be necessary to employ orthogonal technologies.

**Table 1:**
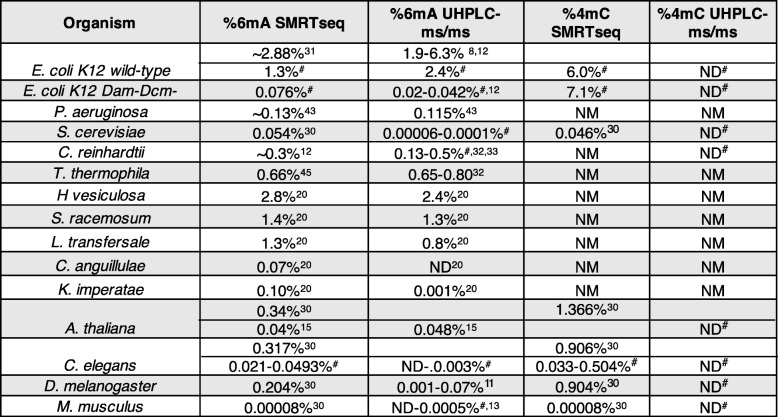
Comparison of SMRTseq concentrations to UHPLC-ms/m. Calculated DNA methylation concentrations from SMRTseq or UHPLC-ms/ms demonstrates that 6 mA quantification by SMRTseq is more accurate at higher levels of 6mA than when it is less abundant and SMRTseq is inaccurate when identifying 4mC

# indicates measurements performed in this study, ND indicates not detectable at our limit of detection (< 0.00003% for 6 mA and < 0.00005% for 4mC), NM indicates not measured or not reported

### SMRTseq overestimates concentrations of 6mA and 4mC in eukaryotes relative to UHPLC-ms/ms

Based on these results, we reviewed SMRTseq data we had generated using DNA extracted from the L4 stage of *C. elegans* [10] and publicly available SMRTseq data generated by Pacbio (http://datasets.pacb.com.s3.amazonaws.com/2014/c_elegans/list.html) of mixed stage *C. elegans* to determine whether SMRTseq 6mA identification accuracy decreased when 6mA abundance is low. Neither of the *C. elegans* datasets completely covered the *C. elegans* genome, but there was sufficiently high coverage to identify 6mA and 4mC modifications provisionally. Our L4 stage SMRTseq dataset had 28.3% genome coverage, while the mixed stage dataset had 81.4% genome coverage, using 25x coverage density as a cutoff. In L4 staged worms, using the strictest calling criteria, 4095 cytosines and 8494 adenines were identified as 4mC and 6mA, respectively – corresponding to 0.039 and 0.046% of the total covered nucleotides (Additional file 4). The mixed stage dataset, which had much higher coverage density, identified a higher frequency of putative 4mC and 6mA sites – 169,497 cytosines and 43,139 adenines were called as methylated by the SMRTseq algorithm (0.588 and 0.081%, respectively) (Additional file 4). There was relatively little overlap between the two datasets (336 shared 4mCs and 278 shared 6mAs of nucleotides covered with sufficient density, Additional file 4), although this overlap was significant (*p* < 2.2E-16 Fisher’s exact test). This difference in methylated sites identified by SMRTseq in L4 and mixed stage *C. elegans* suggests that 4mC and 6mA could either be largely artifactual or, if present, may vary according to developmental stage and/or environmental conditions. However, the frequency of 4mC and 6mA in the SMRTseq analysis far exceeded the frequency detected by UHPLC-ms/ms (Table 1: SMRTseq 6mA: 0.021–0.0493%, UHPLC-ms/ms 6mA: ND-0.003%, SMRTseq 4mC: 0.033–0.504%, UHPLC-ms/ms 4mC: ND).

SMRTseq analysis of called methylated residues in *C. elegans* datasets revealed regional rather than base pair level of overlap of methylation. There was a higher concordance between the two datasets as the length of the sequences being compared increased (27.7% overlap within 50 base pair window, 41.2% within 100 bp, 57.1% within 200 bp, and 77.4% within 500 bp), suggesting that there might be regions that are more prone to methylation. In our previous analysis of 6mA sites in *C. elegans*, we merged two SMRTseq datasets (L4 and mixed stage) to achieve higher sequencing depth and concluded that 6mA was uniformly distributed across the *C. elegans* genome [10]. However, these 6mA datasets were generated with different chemistries, but merged and analyzed as though produced with a single type of chemical reaction. Merging data obtained with different protocols on differently staged worms caused loss of information of genomic patterning. A reanalysis of the same datasets, analyzed separately, now found that both 6mA and 4mC were significantly depleted from the X chromosome, enriched on introns, and depleted from exons (data not shown). However, as stated earlier, the absolute concentration of 6mA and 4mC was significantly higher than what was detected by UHPLC-ms/ms (Table 1), which calls into question the SMRTseq analysis. These analyses suggest that the putative sites of 6mA and 4mC identified by SMRTseq in an organism with a low abundance of these modifications could overestimate true methylation sites.

To determine whether the differences in SMRTseq and UHPLC-ms/ms values we observed in *C. elegans* were present in other eukaryotes, we examined published SMRTseq values in a variety of eukaryotes and compared them with our own and published UHPLC-ms/ms measurements (Table 1). For a variety of fungi, the two methods showed reasonable concordance of 6mA in genomes with abundant 6mA but orders of magnitude more 6mA were reported by SMRTseq than UHPLC-ms/ms in fungi with sparse 6mA [20]. *T. thermophila, L. transfersale, S. racemosum,* and *H. vesiculosa* had SMRTseq 6mA values of 0.66, 1.3, 1.4, and 2.8%, respectively, with similar UHPLC-ms/ms measurements of 0.65–0.8, 0.8, 1.3, and 2.4%, respectively. However, in fungi with less abundant 6mA, such as *K. imperatae* and *C. anguillulae,* SMRTseq values for 6mA abundance were much greater than UHPLC-ms/ms values (SMRTseq 0.1% vs UHPLC-ms/ms 0.001 and 0.007% vs not detectable, respectively). One group reported similar values for 6mA in *A. thaliana* by SMRTseq (0.04%) and UHPLC-ms/ms (0.048%) [15], but a second group detected 10x higher 6mA by SMRTseq (0.34%) [30]. The reported 6mA concentration in *D. melanogaster* by SMRTseq analysis performed by one group (0.204%) [30] was substantially higher than the UHPLC-ms/ms values detected by an independent group (0.001–0.07%) [11]. An analysis of mouse 6mA levels by SMRTseq (0.00008%) reported by [30] lies within the range that we detected by UHPLC-ms/ms and by others [13] in *M. musculus* tissues and cell lines (not detected-0.0005%). Therefore, some SMRTseq analyses of 6mA in different species are in concordance with UHPLC-ms/ms measurements, while other estimates are many folds greater, suggesting that differences in sample preparation, sequence-specific sources of false positive signals, other DNA modificaitons, or bioinformatics analysis could contribute to overestimation of 6mA abundance.

4mC was detected by SMRTseq in *S. cerevisiae* (0.046%), *A. thaliana* (1.366%), *D. melanogaster* (0.904%), and *M. musculus* (0.00008%) [30]. However, we were unable to detect 4mC in any of those species by UHPLC-ms/ms, where our limit of detection was < 0.00005%. The detection of 4mC in eukaryotic gDNA by SMRTseq may be prone to false-positive detection, as we found in our analysis of bacterial DNA. Thus, SMRTseq identification of 4mC and 6mA in both eukaryotes and prokaryotes sometimes greatly exceeds the frequency of these modifications detected by UHPLC-ms/ms (Table 1).

### Exogenous methylated nucleosides can be incorporated into mammalian DNA

**Fig. 5:**
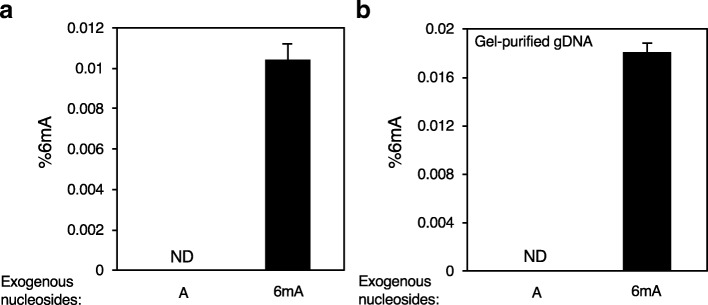
Exogenous methylated nucleosides are incorporated into mammalian DNA. **a** Exogenously supplied 6mA nucleosides were incorporated by C2C12 cells as assessed by UHPLC-ms/ms. Cells were continuously supplemented in their media with 1 mM A or 6mA nucleoside for 5 days. ND is not detected at our limit of detection < 0.00003%. This graph represents the mean +/− SEM for three biological replicates where undigested signal was subtracted from digested signal. **b** Gel-purified C2C12 gDNA reveals incorporation of exogenous 6mA when continuously supplemented in the media (1 mM for 5 days). Graph represents the mean +/− SEM for two biological replicates where undigested signal was subtracted from digested signal

Human cells have been reported to take up exogenous N6-adenine methylated nucleotides and incorporate them into their own DNA [25, 34]. Bacteria, which are consumed, as a primary source of food, such as in *C. elegans,* or that co-exist as microbiota in other metazoa, could provide a source of 6mA for eukaryotes through the recycling of bacterial DNA bases. To determine whether exogenous 6mA is incorporated into mammalian DNA, we supplied concentrated exogenous methylated or unmethylated nucleosides in the media of mouse myoblast C2C12 cells for five days and extracted gDNA after rigorous washing. We then performed UHPLC-ms/ms analysis on digested gDNA. In parallel, undigested gDNA was analyzed to quantify the background amount of unincorporated nucleosides that were co-purified, which was subtracted from the measured concentration of methylated nucleosides in digested DNA. No 6mA was detected in DNA harvested from cells whose culture media contained only unmethylated adenine but 0.0104 +/− 0.0008% 6mA was found in gDNA cultured in medium that contained exogenous N6-methylated adenine. Thus C2C12 cells incorporated exogenous 6mA supplied in the media (Fig. 5a). When these experiments were performed 6mA was below our limit of detection under basal conditions, which highlights the variability of 6mA detection from experiment to experiment. To further confirm that the detected 6mA was incorporated into the gDNA of the mammalian cells, we repeated these experiments using gel purified high mass gDNA. Again, we found significant 6mA incorporated into gel-purified C2C12 gDNA that was similar in abundance to the value measured in gDNA that was not gel-purified (0.0180 +/− 0.0008%) (Fig. 5b). This result suggests that a portion of 6mA DNA methylation in animal genomes could be due to incorporation of exogenous methylated bases rather than autonomously regulated, directed methylation.

## Discussion

Reported estimates of the frequency of 6mA and 4mC modifications in genomic DNA from multiple species as determined by UHPLC-ms/ms and SMRTseq analysis vary widely. This study was undertaken to try to understand the source of these discrepancies. We uncovered several potential artifacts that plague the use of both methods that could lead to overestimates of 6mA and 4mC levels, especially when they are rare (<< 1% of base modified). Importantly, the enzymes used to digest gDNA prior to UHPLC-ms/ms analysis can be contaminated with methylated and unmethylated nucleosides (Fig. 1) that presumably co-purified with the DNases and alkaline phosphatases that are commercially available. When we subtracted the background levels of contaminating 6mA and 4mC present in the enzyme preparations in the absence of gDNA, we found that estimates of 6mA by SMRTseq and UHPLC-ms/ms in bacteria and highly expressing fungi were comparable and likely accurate (Table 1), but much lower, or in some cases at the detection limit, levels of 6mA were found in more recently evolved eukaryotic genomes under basal conditions. Expressing and purifying these enzymes from cells that lack these modifications or removing the contaminating DNA from the enzyme preparations should greatly reduce this UHPLC-ms/ms background and improve its ability to detect rare methylation events. Since 4mC and 6mA are abundant in bacterial DNA, sample contamination with bacteria in food or microbiota also led to concentration overestimates due to bacterial DNA contamination, as we found here in our analysis of changes in 6mA DNA methylation during zebrafish embryo development (Fig. 4). Mycoplasma contamination could provide a source of elevated 6mA in cell lines. Another potential source of artifactually elevated UHPLC-ms/ms estimates of 4mC could arise from sonication of 5mC-containing DNA that gives rise to a methylated cytosine variant, which has mobility similar to 4mC that we denote mC* (Fig. 3).

We also found that SMRTseq likely overestimates 4mC and 6mA abundance, especially when these modifications are rare (Table 1). Although some groups obtained similar values of 6mA using UHPLC-ms/ms or SMRTseq, other groups report much higher 6mA values in eukaryotes by SMRTseq [15, 20, 45]. We think it is likely that these higher estimates are overestimates. It is unclear whether this might be because of bacterial contamination in sample preparation or might simply require more stringent bioinformatics analysis [47]. Accuracy may be especially a problem when methylation does not consistently occur within a consensus motif.

We did not detect 4mC above background by UHPLC-ms/ms in any of the bacterial or eukaryotic gDNAs we analyzed in this study (Fig. 3). There was no 4mC contamination of the DNase/phosphatase enzyme preparation we used, so the lack of detection was not caused by background noise. This was in contrast to SMRTseq analysis of bacterial and eukaryotic DNA [30], which reported 4mC modifications in the percentile range. This discrepancy between UHPLC-ms/ms readings strongly suggests that SMRTseq calls of 4mC in certain bacteria and other species are largely artefactual. SMRTseq analyses may be misinterpreting another modification as 4mC.

## Conclusions

Our review of the literature and our own data here, coupled with our new UHPLC-ms/ms data that take into account background 6mA in enzyme preparations, suggest that 6mA in eukaryotic genomic DNA that have been studied, with the exception of ciliates, chlamydomonas and some fungi, is rare, occurring at most in the ppm range under basal conditions. The 6mA abundance values we obtained by UHPLC-ms/ms in metazoa are close to our limit of detection (< 0.00003%). We also confirmed previous findings [25, 34] that exogenous 6mA nucleosides can be incorporated into eukaryotic DNA, suggesting that some 6mA might be randomly inserted into gDNA from bacterial DNA decay products. However, we found low, but detectable 6mA above background in samples from multiple tissues of gnotobiotic mice, which eliminated bacterial contamination as a source of 6mA. These results taken together suggest that 6mA is a rare modification in more recently evolved eukaryotic genomes under basal conditions. Although we cannot be certain that it exists or is the product of directed DNA methylation, it may be present and become elevated at specific loci or specific developmental stages [50], or plays roles under certain stress conditions [18, 19, 23]. It will be critical, in the coming years, to identify biological conditions where 6mA becomes appreciably elevated. We strongly recommend using and confirming mycoplasma-free cells in each experiment, developing quantitative accurate sequencing technologies to map and quantify 6mA at single-base resolution in gDNA, coupled with specific probe-based pull-down of DNA loci enriched with 6mA for accurate UHPLC-ms/ms analysis, and identifying enzymes that install or erase these methylation events to understand their biological significance in more recently evolved eukaryotes.

## Methods

### DNA digestion and UHPLC-ms/ms analysis

For digestion reactions using Nuclease P1, 1–5 μg of gDNA was incubated with 1 U of Nuclease P1 (Wako USA) in a buffer containing 10 mM NH_4_OAc pH 5.3, 2 mM ZnCl_2_ at 42 °C overnight. Digestion was followed by adding 3.5 μl of 1 M NH_4_HCO_3_ and 1 μl of Phosphodiesterase I from *Crotalus adamanteus* venom (0.001 U/ μl, Sigma-Aldrich) at 37 °C for 2 h and then by addition of 1 U of alkaline phosphatase from *E. coli* (Sigma-Aldrich) at 37 °C for 2 h. For digestion reactions using S1 nuclease (ThermoScientific), gDNA was first denatured at 95 °C for 5 min. The denatured DNA was then incubated with 1 U of S1 nuclease for 2 h at 37 °C, and dephosphorylated by addition of thermosensitive Fast alkaline phosphatase (FastAP, Thermo Scientific) for 1 h at 37 °C. 1–5 μg of genomic (g)DNA was digested to free nucleosides using 5-15 U of DNA Degradase Plus (Zymo Research) in 25 μl reactions incubated for 2 h at 37 °C. For the preparation of calibration standards, pure dCTP, N4-methyl-dCTP (4mdCTP) or 5-methyl-dCTP (5mdCTP) were also digested with 10 U of DNA Degradase Plus in 25 μl reactions for 2 h at 37 °C. After digestion of samples or pure standards, the volume was brought to 100 μl with ddH2O followed by filtration using 0.22 μm Millex Syringe Filters. 5 μl of the filtered solution was analyzed by LC-MS/MS. The separation of nucleosides was performed using an Agilent 1290 UHPLC system with a C18 reversed-phase column (2.1 × 50 mm, 1.8 m). The mobile phase A was water with 0.1% (v/v) formic acid and mobile phase B was methanol with 0.1% (v/v) formic acid. The gradient and flow rate was optimized to maximize the separation of 4mdC and 5mdC peaks. Online mass spectrometry detection was performed using an Agilent 6470 triple quadrupole mass spectrometer in positive electrospray ionization mode. Quantification was accomplished in dynamic multiple reaction monitoring (dMRM) mode by monitoring the transitions of 228.2 → 112.1 (dC), 242.2 → 126.1 (4mdC/5mdC). dC, 4mdC and 5mdC were quantified using corresponding calibration curves generated with pure standards.

To quantify 6mA concentrations in metazoan gDNA samples, we used pure 2’deoxyadenosine (dA) and N6-methyl-2′-deoxyadenosine (6mA) nucleosides as calibration standards. Digested samples or pure nucleoside standards were diluted to 100 μl with ddH2O and filtered through 0.22 μm Millex Syringe Filters. 5 μl of the filtered solution was injected for LC-MS/MS analysis, and analyzed using the Agilent 1290 UHPLC system with a C18 reversed-phase column (2.1 × 50 mm, 1.8 m). Mobile phase A consisted of water with 0.1% (v/v) formic acid and mobile phase B consisted of methanol with 0.1% (v/v) formic acid. Mass spectrometry detection was performed using an Agilent 6470 triple quadrupole mass spectrometer in positive electrospray ionization mode and data were quantified in dynamic multiple reaction monitoring (dMRM) mode, by monitoring the mass transitions 252.1 ➔136.0 for dA and 266.1➔150.0 for 6mA. The ratio of 6mdA/A in gDNA samples was quantified using calibration curves from serial dilutions of pure 6mA or dA standards. As a negative control in each UHPLC-ms/ms experiment, we included a “mock” digestion reaction, consisting of DNA Degradase Plus and digestion buffer in water, without any added DNA. This control established the background level of 6mA and dA in the reagents, which was subtracted from the values of each gDNA sample. We additionally performed mock extractions using proteinase K and RNase A with water processed through DNA extraction columns and found similar levels of contamination as in our mock reaction with water and the digestion enzymes.

### Worm strains

The N2 Bristol strain was used as the wild-type strain. Worms were grown on *dam*^*−*^*dcm*^*−*^ bacteria (NEB C2925) on standard NGM plates in all experiments.

### Zebrafish husbandry and preparation of gDNA from embryos

Zebrafish were housed at 28.5 °C water tanks using standard maintenance protocols [51]. The wildtype AB zebrafish strain was originally obtained from the Zebrafish International Resource Center. Generation of staged embryos was achieved by paired mating of adult zebrafish (3–24 months of age) in divided tanks to separate males from females. Dividers were removed following mating and egg production was monitored to establish the time of fertilization. Zebrafish embryos younger than 3 days post fertilization (dpf) were either washed three times for 10 min in 50 mL distilled water or manually dechorionated followed by washing in water or sequentially in water, three times in 70% ethanol washes for 20 min and then three times in water. All zebrafish embryos were euthanized by freezing and adult fish were euthanized using 250 mg/l tricaine methane sulfonate (MS-222) in accordance with the guidelines of IACUC animal protocol number 04487.

### gDNA extraction

gDNA samples from *R. temporaria* (liver), *G. gallus* (brain), *B. taurus* (brain), *O. aries* (brain), *R. norvegicus* (brain), *C. porcellus* (brain), *M. mulatta* (brain) and *H. sapiens* (brain) were obtained from Zyagen (Life Sciences). gDNA samples from *D. melanogaster* were obtained from the Drosophila species stock center. Gnotobiotic mouse brain tissues were obtained from the Gnotobiotics, Microbiology and Metagenomics Core of the Harvard Digestive Disease Center. Results for Gnotobiotic mouse tissues are presented in Additional file 3. For extraction of *C. elegans* embryo gDNA, gravid adult worms were washed in sterile M9 buffer and bleached with 5% hypochlorite solution to dissolve and kill adult worms and bacteria and release the embryos. Egg pellets were then subjected to six freeze-thaw cycles in liquid nitrogen and incubated overnight in digestion buffer with proteinase K (Invitrogen PureLink Genomic DNA Mini kit). Digested samples were treated with RNase A and gDNA was column-purified using the Invitrogen PureLink Genomic DNA Mini kit using the protocol for mouse tail gDNA. gDNA from SF9 cells (*S. frugiperda*) and human cell lines (*H. sapiens*), mouse ES cells (*M. musculus*), or *C. reinhardtii* was extracted using the Invitrogen PureLink Genomic DNA Mini kit following the manufacturer’s instructions for isolating gDNA from mammalian cells. gDNA from the three yeast species (*S.pombe, S. japonicas,* and *S. cerevisiae*) was extracted folloring the manusfacturer’s instructions for isolating gDNA from Gram Positive Bacterial Cell Lysates, gDNA from *E. coli* was extracted following instructions for isolating gDNA from Gram Negative Bacteria, and gDNA from mouse tissues (*M. musculus*) or *D. rerio* was extracted following instructions for isolating gDNA from mammalian tissues.

### Bacterial 16S primers

Real-time PCR was performed using 16S rRNA primers 16S F: ACTCCTACGGGAGGCAGCAGT, 16S R: TATTACCGCGGCTGCTGGC, using a serial dilution of *E. coli* DNA as standards. Because 16S copy number varies across bacterial species [37], these PCR reactions demonstrate the presence or absence of prokaryotic DNA, but are not necessarily quantitative.

### Zebrafish DNA specific primers

The relative percentage of zebrafish DNA in embryo samples relative to bacterial DNA was assessed using *D. rerio frrs1b* DNA-specific primers: *frrs1b* F: GAGTTTCCTTGGCTGTG, *frrs1b* R*:* AAATGAAGAAAGGGAGG.

### SMRT-sequencing analysis

Raw data are available from 1) Pacbio public database (http://datasets.pacb.com.s3.amazonaws.com/2014/c_elegans/list.html) and 2) GEO accession numbers GSE66504, GSE86993, and GSE116942. SMRT Analysis 2.3.0 was used to detect base modifications, using *C. elegans* ce10 and *E. coli* K-12 MG1655 as references. The RS_Modification_Detection_and Motif_Analysis.1 protocol was applied; only the unambiguously mapped reads with minimum map QV30 and reads in the modification detection step were used. Putative modifications were detected as positions with 20 or higher modification QV (*p*-value < 0.01). To increase reliability, we removed putative modification sites with less than 25X coverage per strand. Motif discovery was performed using DREME [52] and motif scanning was performed using CENTRIMO [53].

### α-Histone H3 ChIP

Two hundred ninety-three T cells were crosslinked with 1% formaldehyde at room temperature for 10 min followed by the addition of 0.125 M glycine. Cells were washed 3 times with ice-cold PBS and the cell pellet was washed sequentially at 4 °C in LB1 buffer (50 mM Hepes-KOH pH 7.5, 140 mM NaCl, 1 mM EDTA, 10% glycerol, 0.5% NP-40, 0.25% Triton X-100, protease inhibitors) and LB2 buffer (20 mM Tris-Cl pH 7.5, 200 mM NaCl, 1 mM EDTA, 0.5 mM EGTA, protease inhibitors) and resuspended in LB3 buffer (20 mM Tris-Cl pH 7.5, 150 mM NaCl, 1 mM EDTA, 0.5 mM EGTA, 1% Triton X-100, 0.1% Na deoxycholate, 0.1% SDS, protease inhibitors) and sonication for 12 15-s cycles using a Q Sonica sonicator (25–30% output) in an ethanol-ice bath. After 2 rounds of centrifugation, 500 μl of the chromatin supernatant fraction (25 μg DNA) was used for immune precipitation with 5 μg of anti-histone H3 antibody (Abcam ab1791). 50 μl of the chromatin fraction was saved as input. Immunoprecipitation was performed with BSA-blocked Protein G Magnetic Dynabeads (Invitrogen) overnight at 4 °C. The immunoprecipitates were washed twice with LB3, twice with LB3-high salt buffer (500 mM NaCl), 3 times with RIPA buffer (50 mM Hepes-KOH pH 7.5, 0.25 M LiCl, 1 mM EDTA, 0.5% Na deoxycholate, 1% NP-40) and once with TE50 buffer (50 mM Tris-Cl pH 8, 1 mM EDTA). DNA-protein complexes were eluted from the beads twice with Talianidis Elution buffer (70 mM Tris-Cl pH 8, 1 mM EDTA, 1.5% SDS) at 65 °C for 15 min. The input sample was diluted 1/10 with elution buffer. After adding 200 mM NaCl, crosslinking was reversed by incubation overnight at 65 °C, and samples were incubated with 20 μg RNAse for 1 h at 37 °C, followed by the addition of 5 mM EDTA and 40 μg of Proteinase K for 2 h at 42 °C. DNA was extracted once with phenol-chloroform and once with chloroform, followed by the addition of 20 μg glycogen and ethanol precipitation overnight at − 20 °C. After centrifugation, the DNA pellets were washed twice with 70% ethanol and DNA was resuspended in 50 μl ddH2O. Input and anti-H3 IP DNA was digested to nucleosides followed by 6mdA quantification using LC-ms/ms as described above.

### Exogenous nucleoside incubation

C2C12 cells were maintained in DMEM (11995–062, ThermoFisher Scientific) with 10% FBS and antibiotics (100 units/ml penicillin and 100 μg/ml streptomycin) under standard tissue culture conditions. Media was replaced every day and supplemented with 1 mM A or 6mA nucleosides for 5 successive days.

## Additional files

Additional file 1: Representative UHPLC-ms/ms chromatograms demonstrate 6mA signal and mock correction. Representative UHPLC-ms/ms chromatograms displays the 6mA peak in *C. elegans* samples (top panel) and mock digestions (lower panel) when signal was a) detected or b) not detected. Mock digestions are subtracted from sample digestions to calculate percent 6mA. c) 6mA concentrations (pmol) in all replicate samples for Fig. 2d, including the mock digestion reactions (black bars) input DNAs (blue bars) and histone H3 IP’d DNAs (red bars). (PDF 57 kb)

Additional file 2: Quantification of prokaryotic DNA in eukaryotic samples. a) Percentage of bacterial DNA as assessed by real-time RT PCR with 16S rRNA specific primers [54]. Zoomed in plot displayed on the right. Most species tested had less than 1% bacterial contamination. No significant correlation was detected when comparing DNA methylation to bacterial contamination in different eukaryotic species (R^2^ = 0.08859, *p* = 0.32). b) Percentage of zebrafish, bacterial, and unknown DNA reads from input and 6mA IP sequencing experiments previously performed [14]. Some bacterial DNA was detected in all samples and it was enriched after 6mA IP but no developmental trend in bacterial concentrations was detected by sequencing analysis. (PDF 31 kb)

Additional file 3: UHPLC-ms/ms quantification of 6mA in gnotobiotic mouse tissues. UHPLC-ms/ms quantification of 6mA in 10 tissues from gnotobiotic mice demonstrates equivalent levels of 6mA. Each bar represents the mean +/− standard error of the mean for 1–3 independent samples (PDF 41 kb)

Additional file 4: SMRTseq location of 4mC and 6mA in *C. elegans* datasets. SMRTseq performed on L4 or mixed stage *C. elegans* were mapped to the genome and their location, gene name, and gene description are listed. The overlap between different SMRTseq analyses is also presented (XLSX 15767 kb)

## Abbreviations

4mC: N4-cytosine methylation
5mC: C5-cytosine methylation
6mA: N6-adenine methylation
ChIP: Chromatin immunoprecipitation
Dam: DNA adenine methyltransferase
Dcm: DNA cytosine methyltransferase
IF: Immunofluorescence
IP: Immunoprecipitation
SMRTseq: Single molecule real-time sequencing
UHPLC-ms/ms: Ultra-high performance liquid chromatography coupled with mass spectrometry
