# Supplementary figures and images for: Sources of artifact in measurements of 6mA and 4mC abundance in eukaryotic genomic DNA
Source: BMC Genomics. 2019 Jun 3;20:445. doi: 10.1186/s12864-019-5754-6 (PMC6547475; doi:10.1186/s12864-019-5754-6)

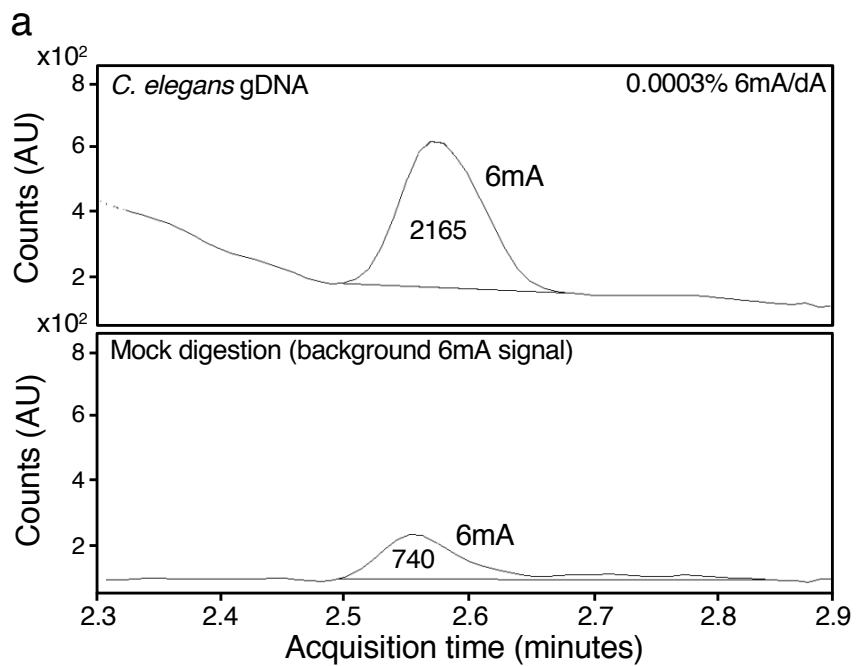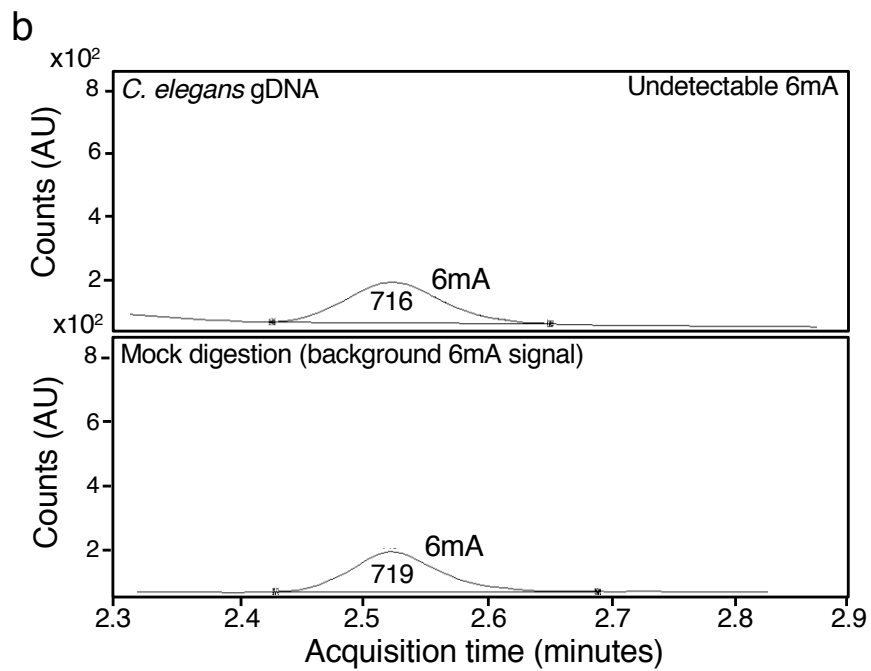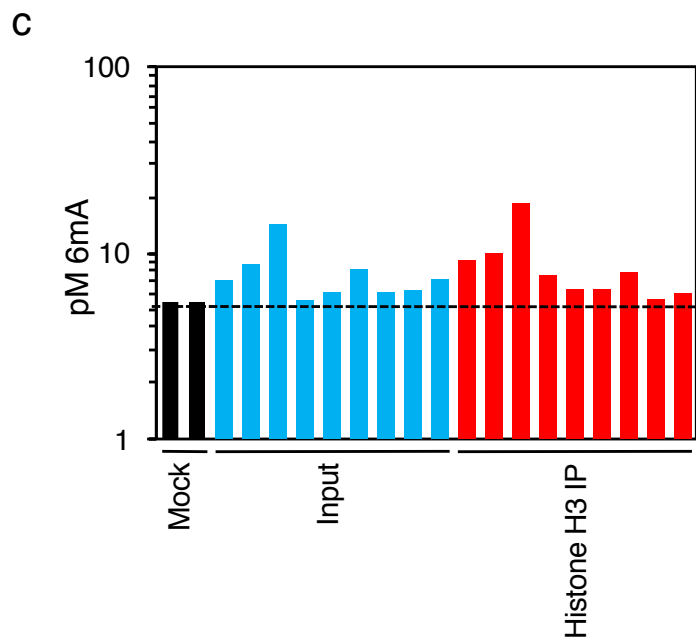

Supplement: Supplementary file 1 — Representative UHPLC-ms/ms chromatograms demonstrate 6mA signal and mock correction. Representative UHPLC-ms/ms chromatograms displays the 6mA peak in C. elegans samples (top panel) and mock digestions (lower panel) when signal was a) detected or b) not detected. Mock digestions are subtracted from sample digestions to calculate percent 6mA. c) 6mA concentrations (pmol) in all replicate samples for Fig. 2d, including the mock digestion reactions (black bars) input DNAs (blue bars) and histone H3 IP’d DNAs (red bars). (PDF 57 kb) [file 12864_2019_5754_MOESM1_ESM.pdf]

a

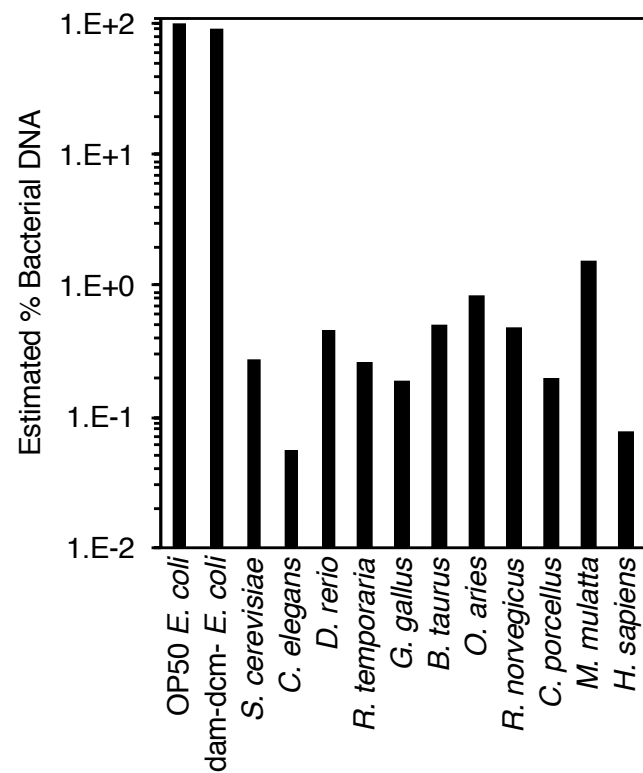

b

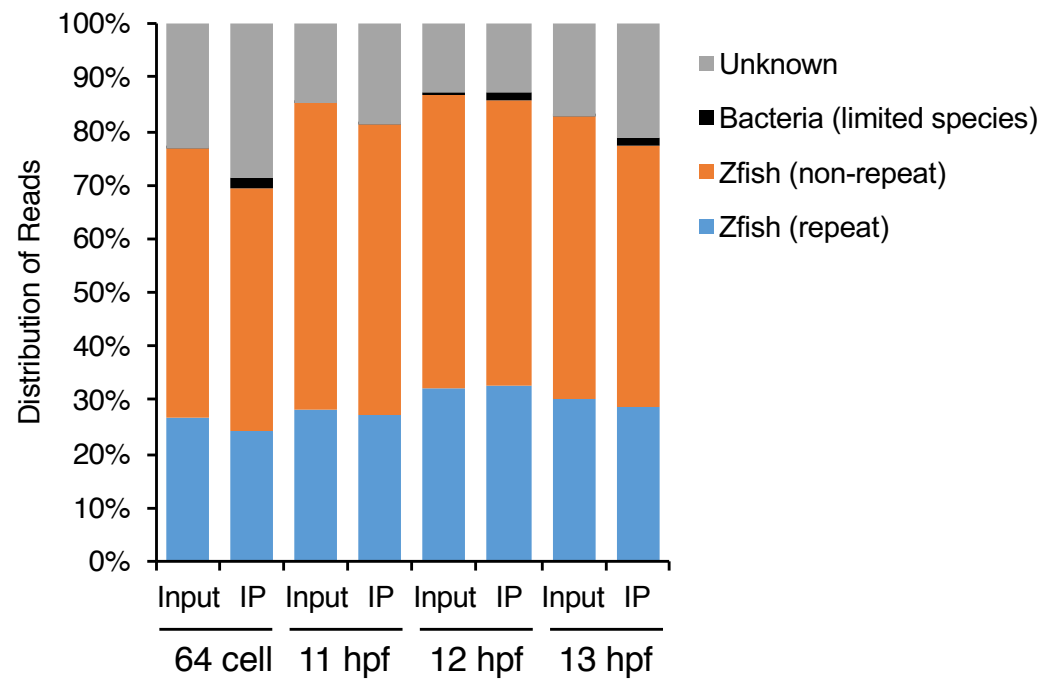

Supplement: Supplementary file 2 — Quantification of prokaryotic DNA in eukaryotic samples. a) Percentage of bacterial DNA as assessed by real-time RT PCR with 16S rRNA specific primers [54]. Zoomed in plot displayed on the right. Most species tested had less than 1% bacterial contamination. No significant correlation was detected when comparing DNA methylation to bacterial contamination in different eukaryotic species (R2 = 0.08859, p = 0.32). b) Percentage of zebrafish, bacterial, and unknown DNA reads from input and 6mA IP sequencing experiments previously performed [14]. Some bacterial DNA was detected in all samples and it was enriched after 6mA IP but no developmental trend in bacterial concentrations was detected by sequencing analysis. (PDF 31 kb) [file 12864_2019_5754_MOESM2_ESM.pdf]

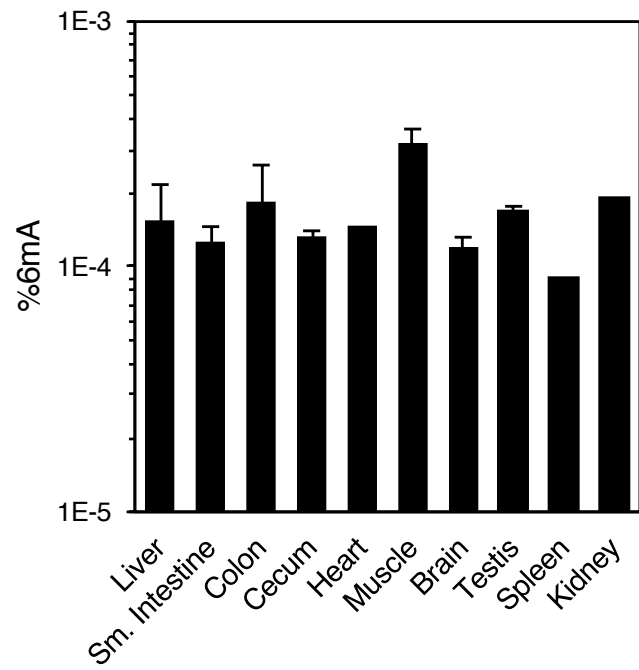

Supplement: Supplementary file 3 — UHPLC-ms/ms quantification of 6mA in gnotobiotic mouse tissues. UHPLC-ms/ms quantification of 6mA in 10 tissues from gnotobiotic mice demonstrates equivalent levels of 6mA. Each bar represents the mean +/− standard error of the mean for 1–3 independent samples (PDF 41 kb) [file 12864_2019_5754_MOESM3_ESM.pdf]
